# Lack of Association of the Caspase-12 Long Allele with Community-Acquired Pneumonia in People of African Descent

**DOI:** 10.1371/journal.pone.0089194

**Published:** 2014-02-26

**Authors:** Jiwang Chen, Esther S. Wilson, Mary K. Dahmer, Michael W. Quasney, Grant W. Waterer, Charles Feldman, Richard G. Wunderink

**Affiliations:** 1 Department of Medicine, Northwestern University, Chicago, Illinois, United States of America; 2 Section of Pulmonary, Critical Care, Sleep and Allergy, University of Illinois at Chicago, Chicago, Illinois, United States of America; 3 Oakbrook Pediatrics, Summerville, South Carolina, United States of America; 4 Department of Pediatrics and Communicable Diseases, University of Michigan, Ann Arbor, Michigan, United States of America; 5 School of Medicine and Pharmacology, University of Western Australia, Perth, Western Australia, Australia; 6 Division of Pulmonology, Department of Internal Medicine, Charlotte Maxeke Johannesburg Academic Hospital, and Faculty of Health Sciences, University of the Witwatersrand, Johannesburg, South Africa; University of Leicester, United Kingdom

## Abstract

Community-acquired pneumonia (CAP) is a common cause of sepsis. Active full-length caspase-12 (CASP12L), confined to the people of African descent, has been associated with increased susceptibility to and mortality from severe sepsis. The objective of this study was to determine whether CASP12L was a marker for susceptibility and/or severity of CAP. We examined three CAP cohorts and two control populations: 241 adult Memphis African American CAP patients, 443 pediatric African American CAP patients, 90 adult South African CAP patients, 120 Memphis healthy adult African American controls and 405 adult Chicago African American controls. Clinical outcomes including mortality, acute respiratory distress syndrome (ARDS), septic shock or severe sepsis, need for mechanical ventilation, and *S. pneumoniae* bacteremia. Neither in the three individual CAP cohorts nor in the combined CAP cohorts, was mortality in CASP12L carriers significantly different from that in non-CASP12L carriers. No statistically significant association between genotype and any measures of CAP severity was found in any cohort. We conclude that the functional CASP12L allele is not a marker for susceptibility and/or severity of CAP.

## Introduction

Even in the antibiotic era, pneumonia remains the most common infectious cause of death worldwide, occurring in all age groups. Among children under five years old, pneumonia is one of the leading causes of death globally accounting for approximately 2 million deaths per year [1]. In addition, pneumonia is the most common source of severe sepsis. The estimated financial cost of treating pneumonia in the USA alone exceeds 12 billion dollars per year [2].

Clinical manifestations of pneumonia are mediated largely by cytokines and chemokines, whose production and release are modulated by caspases, including caspase-12 [3]. Two human isoforms of caspase-12 exist: a full-length caspase-12 (CASP12L) and a truncated form [CASP12S] associated with the C125T polymorphism, which results in a stop codon in exon 4 [4], [5]. CASP12L is confined to approximately 20–25% of people of African descent and has been reported to confer hypo-responsiveness to lipopolysaccharide (LPS)-stimulated cytokine production [5]. Clinically, CASP12L has been associated with increased susceptibility to, and mortality from, severe sepsis [5]. Studies performed in mice also suggest that the long form of caspase-12 is associated with mortality as mice without caspase-12 have a significantly higher survival rate in a murine model of polymicrobial peritonitis sepsis [6].

Mechanistically, CASP12L dampens the production of the pro-inflammatory cytokines IL-1β, IL-18 and interferon-γ by inhibiting caspase-1 activity, which are required for IL-1β and IL-18 maturation [7]. As a consequence, the innate immune response of CASP12L positive individuals apparently fails to control early bacterial replication, so that bacteria invade the bloodstream in overwhelming numbers, resulting in severe sepsis.

Whether individuals with CASP12L have increased susceptibility to pneumonia is unknown. Since pneumonia, both community- and hospital-acquired, is the main source of sepsis [8]–[11], we hypothesized that CASP12L patients would be more susceptible to CAP and more likely to have severe CAP. To test our hypothesis, we analyzed both adult and pediatric CAP cohorts of persons of African descent to determine if an association existed between the CASP12L allele and susceptibility to CAP, as well as clinical outcomes of CAP, including mortality and respiratory failure.

## Materials and Methods

### Memphis adult CAP cohort and controls

A cohort of adult CAP patients (≥18 yrs) admitted to Methodist University Hospital in Memphis between 1998–2001 was recruited. Informed consent was obtained from all patients, and the institutional review board of Methodist Healthcare approved the study. Inclusion criteria and exclusion criteria for this cohort have been reported previously [12]. Mechanical ventilation (MV) was defined as any period of mechanically assisted ventilation via an endotracheal or nasotracheal tube. Patients intubated during a cardiopulmonary arrest, but extubated immediately after successful resuscitation, were not considered as having MV [12]. Septic shock in adults was defined as a systolic blood pressure of<90 mmHg for ≥30 mins in the absence of any other causes of shock and ≥4 hrs of inotropic support after adequate fluid replacement [12]. Septic shock or severe sepsis had to occur within 48 hrs of presentation to the hospital for the patient to be classified as having this end point. Acute respiratory distress syndrome (ARDS) was defined as bilateral infiltrates on chest radiography, a PaO_2_/F_i_O_2_≤200, and no evidence of left atrial hypertension [13]. All patients were evaluated by a pulmonary physician and patients who did not meet a strict definition of CAP were excluded. DNA was available for 241 of the 243 African American CAP patients in the original cohort. The healthy control population consisted of African American blood donors from the same geographical region.

### South African adult CAP cohort

Adult patients (≥18 yrs) admitted to the Charlotte Maxeke Johannesburg Academic Hospital with an admission diagnosis of CAP were recruited between November 3, 2007 and October 3, 2010. Written informed consent was obtained from all patients, and the Human Research Ethics Committee of the University of the Witwatersrand approved this study. All patients had signs, symptoms, and radiographic findings compatible with CAP; all diagnoses were confirmed by a pulmonary physician. Patients were classified as having acute hypoxemia based on an arterial saturation<90% on room air. Blood from each patient was saved onto two Schleicher & Schuell 903 paper cards (Schleicher & Schuell BioScience, Inc, Keene, New Hampshire). In each of five blood circles located on the blood card, 50 µl of blood was deposited. Samples were well separated from each other by sealing each card in a zipped plastic bag. DNA from 90 CAP patients from the South African adult CAP cohort was available for this study. Permission to export human biological specimens from South Africa was approved by the Department of Health, South Africa. Endorsement to import and use these human blood cards was approved by the Northwestern University Institutional Review Board.

### Chicago NUgene African American healthy controls

The NUgene project (http://www.NUgene.org) at Northwestern University is a DNA and clinical information repository designed for genetic studies. As of June 11, 2013, 10564 human DNA samples are in the repository. DNA samples from 405 volunteers of African-descent were randomly selected from this repository.

### Pediatric CAP cohort

The pediatric CAP cohort was prospectively recruited from patients presenting to the Emergency Department or admitted to Le Bonheur Children's Medical Center in Memphis, TN, Children's Memorial Hospital in Chicago, or Children's Hospital of Wisconsin as part of an NIH-sponsored study (NICHD, Genetic Polymorphism in Pediatric Lung Injury). The institutional review boards from each institution approved the study. Inclusion criteria and exclusion criteria were detailed previously [13]. All three hospitals are the major pediatric hospitals in large metropolitan areas and referral centers for a broader area, ensuring representation of the breadth of pediatric CAP. The major end points examined in the cohort in this study were need for mechanical ventilation, acute lung injury (ALI) or ARDS, and mortality rate. ARDS was defined as previously described. ALI was defined as the presence of bilateral infiltrates on chest radiography, a PaO_2_/F_I_O_2_≤300, and no evidence of left atrial hypertension [13]. From this study, the DNA of 443 African American patients were available for analysis.

### DNA isolation and caspase-12 C125T SNP Genotyping

DNA was isolated from whole blood for the Memphis adult CAP cohort, the pediatric CAP cohort, Memphis healthy controls and Chicago NUgene controls. DNA isolation from blood cards in South African CAP cohort was performed using DNAzolBD reagent (DN131, Molecular Research Center Inc) [14]. Genotyping of the caspase-12 C125T SNP (rs497116) for all US African American controls and CAP cohorts was performed with a Taqman genotyping assay [Applied Biosystems Inc (ABI), Foster City, CA]. The specific genotyping assay ID number for caspase-12 C125T is C_2411553_20. To confirm different genotypes, three samples from each genotype were sequenced on both strands. Sequencing data confirmed our Taqman genotyping data. Samples from the South African CAP cohort were genotyped by PCR-RFLP (restriction fragment length polymorphism) with SfaN1 digestion enzyme and two primers for amplification [forward: 5′-GTCATTCTGTGTGTATTAATTGC-3′; reverse: 5′-CCTATAATATCATACATCTTGCTC-3′]. Taqman genotyping of random specimens confirmed results. The PCR-RFLP method for CASP12 C125T SNP genotyping is first reported here and demonstrated in Figure S1.

### Statistical analysis

Allelic frequency, Hardy-Weinberg equilibrium, susceptibility, odd ratios and association between genotyping data and clinical outcome were analyzed by PLINK software [15], [16]. Significance of trends was assessed using chi-square analysis. All *p*-values shown are two-tailed, with a value of<0.05 considered significant.

## Results

### Demographics

The demographics of 241 adult African American patients, 90 adult South African patients and 443 pediatric African American patients with CAP are listed in Table 1. The median age was 49.0 yrs (range, 17–98 yrs) in adults and 25.3 months (range, 0.5–226.6 months) in children. In the Memphis adult CAP cohort, twenty one (8.7%) of 241 patients enrolled required mechanical ventilation, three (1.2%) patients had ARDS, and 10 (4.1%) had septic shock. Twelve (5.0%) deaths occurred in this cohort. In the South African adult CAP cohort of 90 patients, four (4.4%) deaths occurred, five (5.6%) patients required mechanical ventilation, 16 (17.8%) patients had acute hypoxemia, 58 (64.4%) patients had HIV infection, and 41 (45.6%) patients were bacteremic with *S. pneumoniae*. In the pediatric CAP cohort of 443 patients, forty-one (9.3%) patients required mechanical ventilation, 28 (6.3%) had ALI/ARDS, 17 (3.8%) had severe sepsis, 15 (3.3%) patients required vasopressors, 11 (2.5%) had renal dysfunction, and 9 (2.0%) had hematologic dysfunction (Table 2). Only 5 deaths (1.1%) occurred in the pediatric CAP cohort.

**Table 1:** Demographics of patients.

	Memphis CAP	Pediatric CAP	African CAP
Total number	241	443	90
Age (range*)	49.0 yrs (17–98)	25.3 months (0.5–226.6)	36 yrs (20–76)
Gender			
Male (%)	106 (44.0)	238 (53.7)	37 (41.1)
Female (%)	135 (56.0)	205 (46.3)	53 (58.9)
Mortality (%)	12 (5.0)	5 (1.1)	4 (4.4)
Septic Shock (%)	10 (4.1)	17 (3.8)	5 (5.6)
MV (%)	21 (8.7)	41 (9.3)	5 (5.6)
ALI/ARDS (%)	3 (1.2)	28 (6.3)	NA

CAP, community-acquired pneumonia; MV, requirement for mechanical ventilation; ALI, acute lung injury (only for pediatric CAP cohort); ALI/ARDS, acute lung injury/acute respiratory distress syndrome; NA – not assessed; * range of Age: median (minimum, maximum).

**Table 2:** Frequency of caspase-12 C125T genotypes in pediatric community-acquired pneumonia patients related to various clinical outcomes.

CASP12 C125T	CC	CT	TT	*p*-value
Total number (%)	12 (2.7)	120 (27.1)	311 (70.2)	
Mortality (%)	0	1 (0.8)	4 (1.3)	0.59
Severe sepsis (%)	2 (16.7)	4 (3.3)	11 (3.5)	0.24
MV (%)	2 (16.7)	10 (8.3)	29 (9.4)	0.83
ALI/ARDS (%)	2 (16.7)	7 (5.8)	19 (6.1)	0.48
Vasopressor (%)	2 (16.7)	4 (3.3)	9 (2.9)	0.12
Renal dysfunction (%)	1 (8.3)	3 (2.5)	7 (2.3)	0.42
Hematologic dysfunction (%)	1 (8.3)	2 (1.7)	6 (2.0)	0.50
Asthma (%)	3 (25)	19 (15.8)	62 (19.9)	0.58
BPD (%)	0	7 (5.8)	8 (2.5)	0.29
CHD (%)	1 (8.3)	1 (0.8)	10 (3.2)	0.61

MV: mechanical ventilation; ALI/ARDS, acute lung injury/acute respiratory distress syndrome; BPD: bronchopulmonary dysplasia; CHD: congenital heart disease.

### Frequency of caspase-12 C125T SNP

The distribution of caspase-12 C125T genotypes is shown in Table 3. All groups were in Hardy-Weinberg equilibrium. In the African American CAP cohorts and controls, genotypic frequencies of CC (CASP12L homozygotes), CT (heterozygotes) and TT (CASP12S homozygotes) were comparable in the Memphis CAP and pediatric CAP cohorts as well as the two control populations. Between 24.1%–29.8% people carried the CASP12L allele in these African American cohorts and controls, similar to the frequency in other populations of African Americans (5). Compared to the Memphis adult and NUgene controls, the CASP12L variant was not more frequent in either the Memphis adult CAP (*p* = 0.63) or pediatric CAP cohorts (*p* = 0.16). However, in the South African CAP cohort, 45.6% carried the CASP12L allele, significantly higher than in either controls or CAP patients in the US cohorts (*p*<0.001).

**Table 3:** Frequency of caspase-12 genotypes in adult, African and pediatric community-acquired pneumonia and healthy control populations.

CASP12 C125T	CC	CT	TT
Memphis Adult CAP (%)	6 (2.5)	52 (21.6)	183 (75.9)
Pediatric CAP (%)	12 (2.7)	120 (27.1)	311 (70.2)
South African Adult CAP (%) *	12 (13.3)	29 (32.2)	49 (54.4)
Memphis healthy controls (%)	3 (2.5)	28 (23.3)	89 (74.2)
Chicago NUgene controls (%)	12 (3.0)	92 (22.7)	301 (74.3)

CAP: community-acquired pneumonia; CC: CASP12L homozygotes; CT: CASP 12L heterozygote; TT: CASP12S homozygotes; * In the South African CAP cohort, 45.6% carried the caspase-12 C allele (CASP12L), significantly higher than in either controls or CAP patients in the US cohorts (*p*<0.001).

### Lack of Association of the caspase-12 long allele with severity of CAP

Association of the caspase-12 long allele with severity of CAP was examined in the pediatric and adult cohorts (Table 2, 4, 5). No deaths occurred in CASP12L homozygotes in either the adult or pediatric CAP cohorts. In the Memphis CAP cohort, only one CASP12L heterozygote death (1.9%) occurred in 52 adult CAP patients, with one CASP12L heterozygote death (0.8%) in 120 pediatric CAP patients. In the South African adult CAP cohort, one death (8.3%) occurred in 12 CASP12L homozygotes, 2 deaths (6.9%) in 29 CASP12L heterozygotes and 1 death (2.0%) in 49 CASP12S homozygotes (*p* = 0.44). Thus, mortality was not associated with CASP12L genotype in either cohort alone. Even after combining the adult and pediatric cohorts, mortality in CASP12L heterozygotes and homozygotes was not significantly different than that in CASP12S homozygotes (2.3% vs 5.2%, *p* = 0.12).

**Table 4:** Frequency of caspase-12 C125T genotypes in the Memphis adult community-acquired pneumonia patients related to various outcomes.

CASP12 C125T	CC	CT	TT	*p*-value
Total number	6	52	183	
Mortality (%)	0	1 (1.9)	11 (6.0)	0.18
Septic shock (%)	0	1 (1.9)	9 (4.9)	0.27
MV (%)	1 (16.7)	3 (5.8)	17 (9.3)	0.78
ARDS (%)	0	0	3 (1.6)	0.34

MV: mechanical ventilation; ARDS: acute respiratory distress syndrome.

**Table 5:** Frequency of caspase-12 C125T genotypes in South African community-acquired pneumonia patients related to various clinical outcomes.

CASP12 C125T	CC	CT	TT	*p*-value
Total number (%)	12 (13.3)	29 (32.2)	49 (54.4)	
Mortality (%)	1 (8.3)	2 (6.9)	1 (2.0)	0.44
Shock (%)	0	3 (10.3)	2 (4.1)	0.34
MV (%)	0	3 (10.3)	2 (4.1)	0.34
Acute hypoxemia (%)	2 (16.7)	4 (13.8)	10 (20.4)	0.37
Renal dysfunction (%)	1 (8.3)	1 (3.4)	1 (2.0)	0.56
HIV (%)	9 (75.0)	21 (72.4)	28 (57.1)	0.70
Blood *S.pneumoniae* positive (%)	6 (50.0)	13 (44.8)	22 (44.9)	0.92
Diabetes (%)	1 (8.3)	2 (6.9)	1 (2.0)	0.48

MV: mechanical ventilation.

No statistically significant association between genotype and any additional measures of CAP severity was found for any cohort. In the Memphis adult CAP cohort, a trend toward a higher frequency of septic shock in patients without the CASP12L allele compared to the patients with at least one CASP12L allele was observed (Table 4, 4.9% vs 1.7%, *p* = 0.27). Need for mechanical ventilation was no more frequent in adult CAP patients without the CASP12L allele compared to patients with the CASP12L allele (Table 4, 9.3% vs. 6.9%, *p* = 0.78). In the pediatric CAP cohort, approximately 4.5% of patients with at least one CASP12L allele developed severe sepsis, a frequency similar to patients without the CASP12L allele (Table 2, 3.5%, *p* = 0.24). Approximately 9.1% of pediatric CAP patients with one or more copies of the CASP12L allele needed mechanical ventilation, which is not significantly different from the frequency for patients who are CASP12S homozygotes (Table 2, 9.4%, *p* = 0.83). In the pediatric CAP cohort, no association was found between the CASP12L allele frequency and other clinical phenotypes, such as asthma, history of bronchopulmonary dysplasia and congenital heart disease (Table 2). In the South African adult CAP cohort, 19/41 (46.3%) of *S. pneumonia* bacteremia cases had at least one CASP12L allele, however this rate was not significantly higher than that seen in the patients who were CASP12S homozygotes (46.3% *vs* 44.9%, *p* = 0.92, Table 5). The frequency of other clinical phenotypes, including shock, need for mechanical ventilation, ALI/acute hypoxemia, renal dysfunction and HIV infection, were not significantly different between the CASP12L and the CASP12S groups (Table 5).

## Discussion

This analysis is the first to examine any association between human full-length, active caspase-12 [CASP12L] and either susceptibility to or severity of CAP. In both adult and pediatric African American CAP patients, we found no association of CASP12L allele with mortality, need for mechanical ventilation, ALI/ARDS, septic shock/severe sepsis or *S. pneumoniae* bacteremia.

These results were unexpected as the study of Saleh et al showed that CASP12L was associated with sepsis mortality in individuals of African descent and hyporesponsiveness to lipopolysaccharide-stimulated cytokine production *ex vivo* [5]. Studies performed on caspase-12 deficient mice and their wild-type siblings have also indicated that CASP12L is associated with adverse outcomes from sepsis [6]. Interestingly, a recent study by Ferwerda et al showed conflicting results, with no association of the CASP12L variant in African individuals with cytokine response to LPS or *Yersinia pestis*, a Gram-negative bacterium [17].

Several explanations for the observed lack of association of CASP12L with either mortality or CAP severity exist. One possibility is that the CASP12L variant has pathogen-specific effects. Other researchers have observed that associations between genetic polymorphisms can differ depending on whether the infecting agent is a Gram-positive or Gram-negative pathogen [18], [19]. CAP etiology is dominated by Gram-positive bacteria (*Streptococcus pneumoniae* and *Staphylococcus aureus*) [20], atypical intracellular pathogens (*Chlamydophila* and *Mycoplasma* spp.), and viruses, with a much smaller role for Gram-negative pathogens. In contrast, the initial study examining association with sepsis recruited patients from a surgical intensive care unit with a higher probability of Gram-negative pathogens [5]. In the South African CAP cohort, 46.3% patients carrying the CASP12L allele were *S. pneumoniae* positive compared to 44.9% of CASP12S homozygous patients, which was not significantly different. This possibility was further supported by our mouse model of *S. pneumoniae*-mediated pneumonia in which caspase-12 deficient mice did not have a survival benefit (unpublished data), compared to a Gram-negative and anaerobe dominated peritonitis model [5].

Another possible explanation for the apparent discrepancy between our results and those of Ferwerda et al [17] and the initial report by Saleh et al [5] may have to do with the gender of the study subjects. Mouse models demonstrate gender disparity in human CASP12L expression and innate immunity to *Listeria mococytogenes* infection [21]. If expression of the human CASP12L variant is different in males and females, as suggested by work in the mouse model, differences in the frequency of males in the various study cohorts could have resulted in conflicting results or differences in the degree of the effect.

Lastly, the number of individuals who died in our cohorts was relatively small, as was the number of individuals with septic shock/severe sepsis. If the effect of the CASPL variant was less than originally assumed, we may not have been able to detect an effect.

In the South African CAP cohort we studied, 45.6% patients carry the CASP12L allele, which is significantly higher than the percentage (21.6%, *p*<0.001) in the South African population reported by Saleh et al [5]. Whether this difference in CASP12L allele frequency is related to susceptibility to pneumonia is unclear. Differences may simply reflect sampling different subpopulations since Xue et al [22] reported that in some African populations up to 60% of individuals carry the CASP12L allele.

The advantage of maintaining the CASP12L allele during human evolutionary history is unclear. The C125T polymorphism underwent rapid positive selection such that all racial and ethnic groups are nearly uniformly CASP12S homozygote except for approximately 20% of people of African descent [22]. In our study, 24.1–29.8% of African Americans still carry CASP12L, despite removal from whatever environmental selection force may have maintained its presence in African populations. Persistence of the CASP12L in modern individuals of African descent is hard to explain, especially given a survival disadvantage during sepsis [5]. The slightly lower mortality in CASP12L CAP patients is inadequate to explain either its persistence in African-descent populations or the excess CAP mortality in African American populations [23].

### Conclusion

We have demonstrated that the functional CASP12L allele is not associated with disease susceptibility, mortality, or severity in either adult or pediatric patients with CAP.

## Supporting Information

Figure S1 Human caspase-12 C125T SNP genotyping by PCR-RFLP (restriction fragment length polymorphism). Two primers (forward: 5′-GTCATTCTGTGTGTATTAATTGC-3′; reverse: 5′-CCTATAATATCATACATCTTGCTC-3′) were used for PCR amplification. PCR products (314 bp) were digested with SfaN1 enzyme. Taqman genotyping of random specimens confirmed results. Agarose gel electrophoresis (2%) was used to separate PCR-RFLP products. DNA bands were stained by ethidium bromide, and a representative image was taken with a camera under UV light. Each lane represents a sample. Lane 2 is CASP12L homozygote, Lanes 7, 9, 14 are CASP12L heterozygotes, and the rest of the lanes are CASP12S (short truncated caspase-12) homozygotes. The last lane is a 100-bp DNA marker.
(TIF)
