# Supplementary figures and images for: Lack of Association of the Caspase-12 Long Allele with Community-Acquired Pneumonia in People of African Descent
Source: PLoS One. 2014 Feb 26;9(2):e89194. doi: 10.1371/journal.pone.0089194 (PMC3935862; doi:10.1371/journal.pone.0089194)

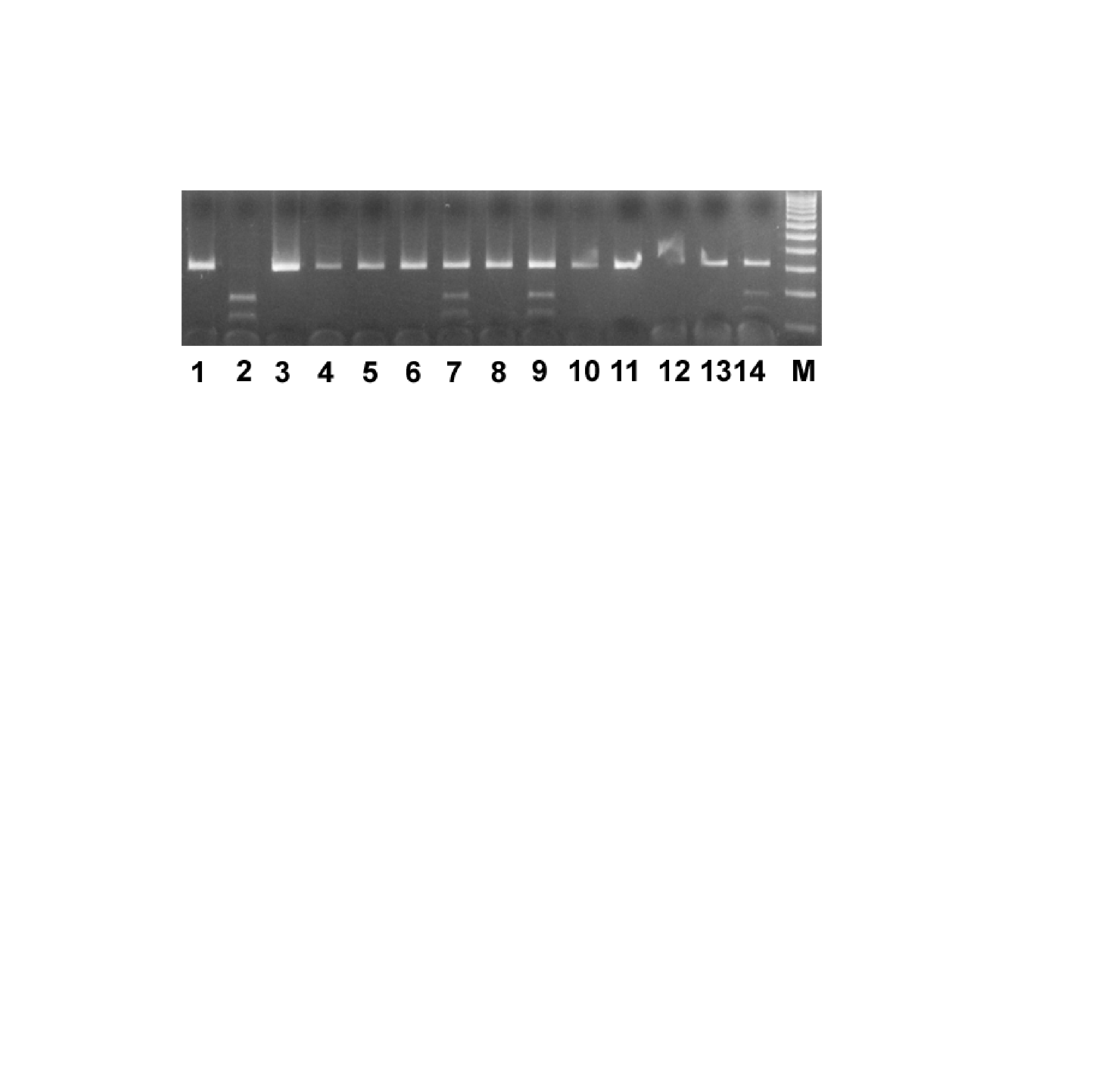

Supplement: Figure S1 — Human caspase-12 C125T SNP genotyping by PCR-RFLP (restriction fragment length polymorphism). Two primers (forward: 5′-GTCATTCTGTGTGTATTAATTGC-3′; reverse: 5′-CCTATAATATCATACATCTTGCTC-3′) were used for PCR amplification. PCR products (314 bp) were digested with SfaN1 enzyme. Taqman genotyping of random specimens confirmed results. Agarose gel electrophoresis (2%) was used to separate PCR-RFLP products. DNA bands were stained by ethidium bromide, and a representative image was taken with a camera under UV light. Each lane represents a sample. Lane 2 is CASP12L homozygote, Lanes 7, 9, 14 are CASP12L heterozygotes, and the rest of the lanes are CASP12S (short truncated caspase-12) homozygotes. The last lane is a 100-bp DNA marker. (TIF) [file pone.0089194.s001.tif]
